# A Spiking Neural Network for Non-Invasive Glucose Estimation on Wearable Bioimpedance Biosensors, with a Multiplication-Free Neuromorphic Path

**DOI:** 10.3390/bios16090469

**Published:** 2026-08-27

**Authors:** Matheus Willian Sprotte, Pedro Bertemes Filho

**Affiliations:** Department of Electrical Engineering, Santa Catarina State University (UDESC), Joinville 89219-710, Brazil; mw.sprotte@edu.udesc.br

**Keywords:** spiking neural networks, bioimpedance, non-invasive glucose estimation, edge AI, neuromorphic regression, embedded systems, ISO 15197, consensus error grid, patient calibration

## Abstract

Wearable glucose monitoring demands low-power local processing, but conventional neural networks rely on energy-intensive multiply–accumulate (MAC) operations that limit battery life. This study shows that a Spiking Neural Network (SNN), built on a regression-adapted Leaky Integrate-and-Fire (LIF) neuron, can estimate blood glucose from multi-frequency bioimpedance and auxiliary biosignals with clinically auditable accuracy at low computational and memory cost. Using data from 98 patients (717 measurements, eGluco3 device, Azambuja Hospital, Brusque, Brazil) evaluated by 5-fold walk-forward cross-validation under ISO 15197:2013, three main findings emerge. First, a new calibration method—the Patient Fingerprint, built from each patient’s first K sensor readings—outperforms conventional one-hot patient encoding (14.2 ± 2.6 mg/dL vs. 15.4 ± 3.3 mg/dL mean absolute error) and, unlike one-hot, requires only these *K* readings rather than the patient’s presence in the training set; a leave-patients-out analysis confirms that the fingerprint captures individual physiology and that unseen-patient accuracy improves with calibration depth but remains clinically insufficient (MAE 94.8→65.9 mg/dL from K=3 to K=5), positioning clinical-grade cross-patient generalization on a larger cohort as the primary scaling axis. Second, the direct-injection fingerprint model reaches 100% of the samples within Consensus Error Grid Zones A+B across all validation folds (the rate-coding variant reaches 98.8%, just below the 99% Criterion B threshold), without requiring any demographic or clinical metadata; sensor history alone renders such records redundant; and Criterion A, however, stays below the 95% normative threshold, so the results support clinical safety rather than formal certification. Third, replacing the analog input encoding with a multiplication-free rate-coding scheme removes all first-layer MAC operations at a cost of 2.7 mg/dL additional error; because the additional microticks raise the total operation count, this defines a design lever whose energy payoff is specific to neuromorphic hardware rather than a net saving on conventional microcontrollers. Together, these results demonstrate that SNNs offer a clinically auditable, self-calibrating, and memory-efficient path to continuous glucose estimation on embedded wearable devices.

## 1. Introduction

The development of wearable technologies for continuous health monitoring requires the integration of biosensors with local computational intelligence. The central topic of this research is the non-invasive estimation of blood-glucose levels using multi-frequency bioimpedance spectroscopy. This multimodal approach yields high-dimensional physiological data since the electrical properties of skin and cell membranes vary significantly across frequency sweeps (e.g., from 100 kHz to 400 kHz) [1,2]. However, deploying real-time predictive models directly on microcontroller-based wearable devices—a paradigm known as Edge AI—imposes severe constraints on hardware and energy consumption [3,4].

The fundamental problem addressed here is the high computational cost of conventional Artificial Neural Networks (ANNs) for this continuous-prediction task. ANNs rely on continuous floating-point data representations and require a massive number of energy-hungry multiply–accumulate (MAC) operations. For continuous monitors powered by small-capacity batteries, this traditional processing scheme leads to rapid energy depletion, precluding long-term autonomous operation [5,6].

To address this problem, we explore Spiking Neural Networks (SNNs) as a bio-inspired alternative for biosignal processing [2]. SNNs incorporate the notion of time into their operational model and communicate through discrete, sparse binary pulses (spikes). This event-driven nature replaces complex multiplications with simple additions, substantially reducing the energy needed for inference at the edge [3,7]. Moreover, although SNNs are widely used for classification, adapting their architecture for continuous regression is a promising challenge for the accurate estimation of biological parameters [8].

The overall objective of this work is to develop and evaluate a regression model based on Spiking Neural Networks capable of processing high-dynamic-range multi-frequency bioimpedance signals for non-invasive glucose estimation, with a primary focus on its computational feasibility for low-power embedded systems. To this end, the study first proposes the preprocessing and normalization of a clinical dataset—comprising bioimpedance measurements and contextual variables—formatted for temporal spike encoding. It then describes an SNN architecture based on the Leaky Integrate-and-Fire (LIF) neuron, which is specifically tuned for continuous regression. Training is performed offline using Backpropagation Through Time (BPTT) together with Surrogate Gradient techniques. Finally, the research evaluates the model’s clinical accuracy using the error criteria defined by the international standard ISO 15197 [9] for glucose-monitoring systems, and it maps its theoretical computational-cost profile to support a future embedded C/C++ implementation with dynamic calibration capabilities [10,11].

## 2. Background and Related Work

This section reviews the theoretical concepts underlying the project and presents recent work on neuromorphic networks for biosignal processing.

### 2.1. Concepts

Multi-frequency bioimpedance spectroscopy consists of the non-invasive measurement of the opposition of biological tissues to an alternating electrical current [12]. Dielectric properties vary dynamically across frequency sweeps (e.g., 100 kHz to 400 kHz), producing high-dimensional data that can be correlated with local metabolic parameters such as glucose.

Spiking Neural Networks (SNNs) are computational models that process information through discrete, sparse temporal events (spikes) [13]. They employ dynamical models such as the Leaky Integrate-and-Fire (LIF) neuron, emitting pulses only when the membrane potential crosses a threshold [14]. This replaces continuous multiplications with asynchronous additions, reducing computational cost.

Edge inference and regression. Edge AI processes data locally on the device, removing the dependence on servers. To adapt SNNs for continuous regression on edge hardware, the firing threshold of the output layer is suppressed, allowing the accumulated membrane potential to be read directly as an analog estimate.

### 2.2. Related Work

Focusing on computational feasibility for biomedical edge devices, Kim and Kim (2024) [1] and Choi (2024) [2] consolidate the algorithmic advantage of SNNs over conventional ANNs for biosignal analysis, showing that the sparse, event-driven nature of spiking networks converts complex MAC operations into simple conditional accumulations (additions). This substantial reduction in arithmetic complexity mitigates rapid battery drain, establishing SNNs as a promising approach for microcontroller implementations in healthcare.

Dealing specifically with non-invasive glucose prediction, Cruz Castañeda and Bertemes Filho (2024) [15] integrated multivariable biosensor data—bioimpedance, SpO_2_ and temperature—into an Edge-IoT model for smart cities. For remote processing, the authors established a broad baseline of twelve AI models, ranging from deep networks (1D-CNN, MLP) and classical approaches (SVR, KNN-R) to ensemble methods (Random Forest, AdaBoost). The best performance was obtained by a CatBoost regressor operating in the Cloud Learning Layer, reaching a test RMSE of 27.97 mg/dL. Although the result validates the clinical efficacy of bioimpedance, stress tests exposed the fragility of the centralized architecture: under a load of 50 concurrent users, the system suffered a severe network bottleneck, with latency peaks of nearly 4 s (3737 ms) and an 11.46% request-failure rate. This delay shows that cloud dependence remains a critical operational barrier, reinforcing the need to move analytical inference entirely onto edge microcontrollers. Furthermore, the predecessor’s evaluation was limited to engineering metrics (MSE, RMSE, R^2^) and did not apply ISO 15197 as a clinical-validation criterion.

To overcome the limitations of edge architectures in analytical tasks, Lee et al. (2025) [8] investigated the feasibility of continuous prediction of local biological parameters. They proposed a model that extracts the spike rate from a neuromorphic network and applies Support Vector Regression (SVR) to continuously estimate heart rate from ECG signals. The results showed that this SNN–regression integration reaches accuracy comparable to conventional deep ANNs at a substantially lower computational cost, although it still depends on a secondary algorithm at the output stage.

Beyond theoretical feasibility, recent literature confirms the practical success of SNNs across biomedical engineering. Chu et al. (2023) [7] addressed accuracy loss in noisy ECG classification: using an SNN classifier enhanced with quantization-aware training, the system overcame raw-signal distortions and reached 98.24% accuracy in arrhythmia detection on wearable devices. Hens et al. (2025) [11] applied SNNs to complex biosignal classification for low-back-pain detection, introducing LAST (Learning Adaptive Spike Thresholds), a trainable encoder that adjusts firing thresholds dynamically, and obtaining over 80% accuracy. Zhao et al. (2023) [3] tackled continuous neural-signal monitoring on portable devices with an event-driven hybrid architecture, achieving accurate classification with extreme energy efficiency (0.99–4.38 μJ per class). Sava et al. (2023) [16] addressed SNN instability when processing high-dynamic-range medical data through recurrent and feed-forward inhibition that compresses and stabilizes physiological data at the network input. Reddy et al. (2024) [17] confronted the non-differentiability of spikes by combining BPTT with surrogate gradients, reaching about 86% accuracy with a very low-complexity architecture. Finally, Martis et al. (2025) [5] developed SYNtzulA, a custom SNN accelerator for near-sensor processing with a consumption of only 36.5 pJ per synaptic operation.

## 3. Materials and Methods

**Figure 1:**
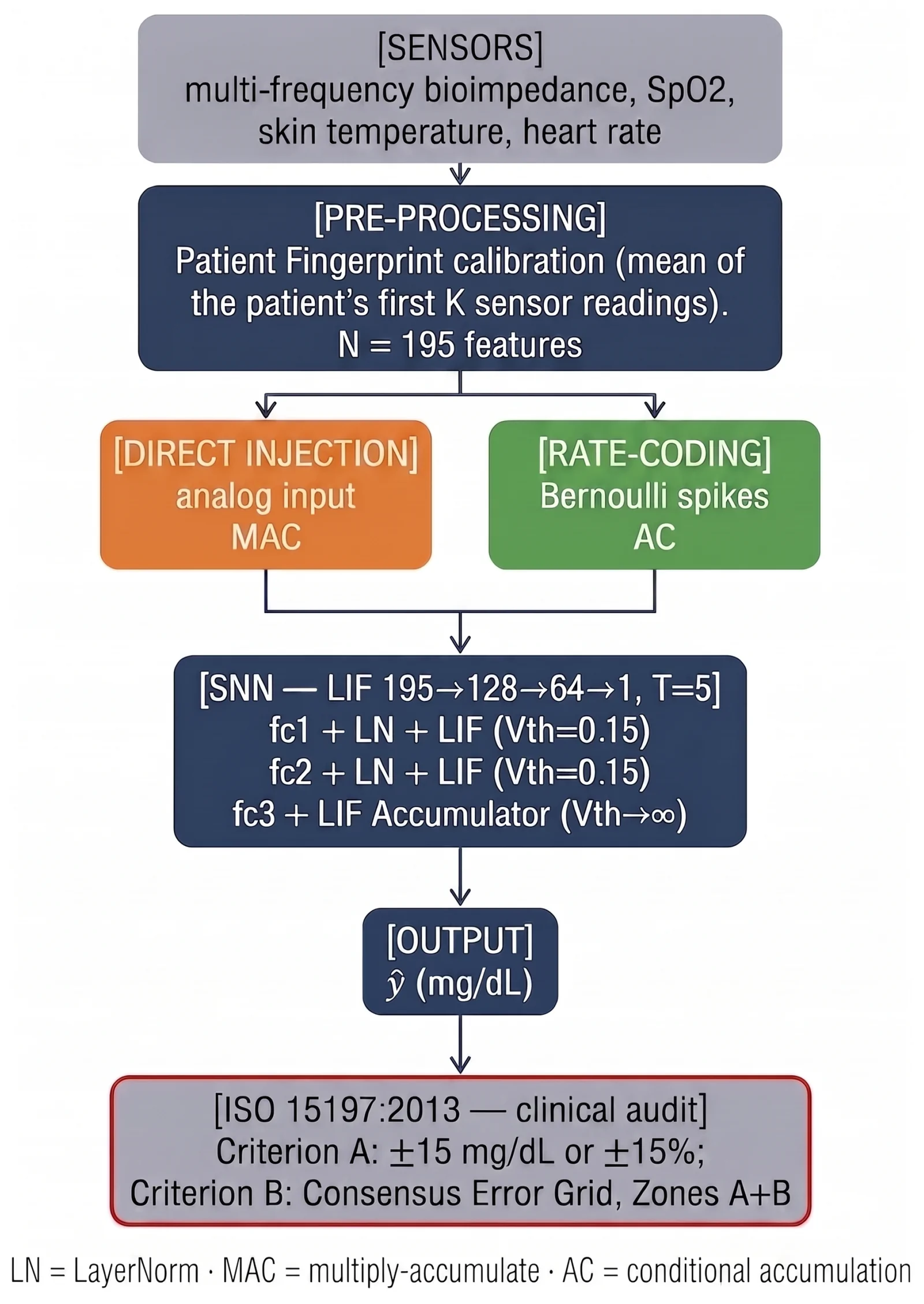
Overview of the proposed system. The flow starts from multimodal sensors (bioimpedance, SpO_2_, skin temperature, heart rate), passes through preprocessing with Patient-Fingerprint calibration (the mean of each patient’s first *K* sensor readings; N=195 features), branches into the two input-encoding topologies—Direct Injection (MAC) and Rate-Coding (AC)—and converges on the SNN-LIF architecture (195→128→64→1,T=5), with LayerNorm and LIF neurons ($V_{th}=0.15$) in the hidden layers and a leak-free accumulator ($V_{th}\to \infty$) at the output. The output $\hat{y}$ (mg/dL) is submitted to the two-phase clinical audit of ISO 15197:2013: Criterion A (±15 mg/dL or ±15%) and Criterion B (Consensus Error Grid, Zones A+B). LN = LayerNorm; MAC = multiply–accumulate; AC = conditional accumulation.

This work proposes a fully spike-based continuous glycemia-estimation architecture designed for autonomous execution on ultra-low-power embedded devices. Figure 1 presents an overview of the proposed system, from multimodal biosignal acquisition to clinical auditing under ISO 15197.

The methodology is structured as a motivated progression of design decisions, in which each stage starts from a limitation identified in the previous one. First, we establish the dataset, the validation protocol and the end-to-end LIF regression topology, exploring alternatives (discrete classification, hybrid SNN+SVR extraction) to justify the architectural choice. We then compare two input-encoding strategies—Direct Injection (MAC) and Rate-Coding (AC)—quantifying the trade-off between clinical accuracy and energy savings. The next stage investigates whether patient clinical-profile data (diabetes type, BMI, sex) improve individual accuracy, revealing that such metadata partially compensate for the absence of individualized physiological calibration. This finding motivated the core mechanism of the solution: the Patient Fingerprint, a calibration vector computed from the patient’s first *K* measurements that replaces one-hot encoding and, by not depending on identities seen in training, in principle enables the calibration of unseen patients. Finally, we evaluate the portability of the trained model to a distinct clinical cohort (ICU2025), delimiting the scope of the solution.

### 3.1. Dataset and Preprocessing

Cohort and acquisition. The study used multisensor monitoring data collected in a controlled clinical environment from two distinct campaigns: ambulatory patients diagnosed with diabetes (2024 campaign) and patients admitted to an intensive-care unit (2025 campaign), with capillary-glucose reference determined by a glucometer. After integrity validation and physiological filtering (steps 1–2 of the pipeline described below), the consolidated base comprises 98 patients and 717 multisensor measurements, concentrated in 2024 (67 patients, 575 measurements) and 2025 (31 patients, 138 measurements). From this base, the analysis cohorts summarized in Table 1 are derived, combining a temporal filter (ambulatory 2024 vs. ICU 2025) with a clinical-profile integrity criterion. The final model is trained and evaluated on the curated ambulatory cohort (sano2024); the ICU cohort serves only as the target domain of the transfer study. Each acquisition simultaneously records five biosignal modalities: (i) multi-frequency bioimpedance—magnitude (*m*) and phase (*f*) measured at 32 distinct frequencies in 2-probe and 4-probe configurations, totaling 32×2×2=128 raw channels; (ii) photoplethysmography (PPG)—minimum and maximum amplitude per acquisition window at 4 wavelengths, yielding 8 optical channels; (iii) pulse oximetry (SpO_2_), 1 channel; (iv) skin temperature, 1 channel; (v) heart and pulse rate, 1 channel.

Patient clinical profile. In addition to the sensor measurements, the database stores a per-patient clinical profile linked by a unique identifier. The available fields are date of birth (converted to age in years), height and weight (from which the body mass index, BMI, is derived), sex (M=1.0; F=0.0), and diabetes type in clinical ordinal encoding (NP=0.0; ND=0.33; T2=0.66; T1=1.0). The integrity criterion required a height between 1.40 and 2.20 m, a weight between 30 and 300 kg and an age between 10 and 100 years. Of the 98 patients, 58 hold a real, valid clinical profile; the remaining 40 lacked a valid record. For the experiments based only on sensors and identity (one-hot), these 40 patients were retained—their clinical fields filled by the median and signaled by a flag—since such models do not consume clinical features. Conversely, any analysis that actually uses clinical features (enrichment and importance) was restricted to the subset of 58 patients with a real profile, avoiding contamination by imputed values.

Sanitization pipeline. Preprocessing followed a four-step sequence: (1) integrity validation—discarding records flagged as invalid by the device firmware (contact failure or motion artifact); (2) physiological filtering—retaining only records with reference glycemia in [50,600] mg/dL (717 measurements from 98 patients after this step)—the broad physiological admissibility range; the champion sano2024 cohort’s reference glucose spans [70,600] mg/dL, which defines the output normalization scaler reused throughout (Section 3.8); (3) cohort filtering—selecting the sano2024 cohort, i.e., patients with at least 80% of measurements in 2024, restricting the study to contemporary, homogeneous data; and (4) zero-variance filtering—removing channels with null variance, leaving 96 active features for the model.

**Figure 2:**
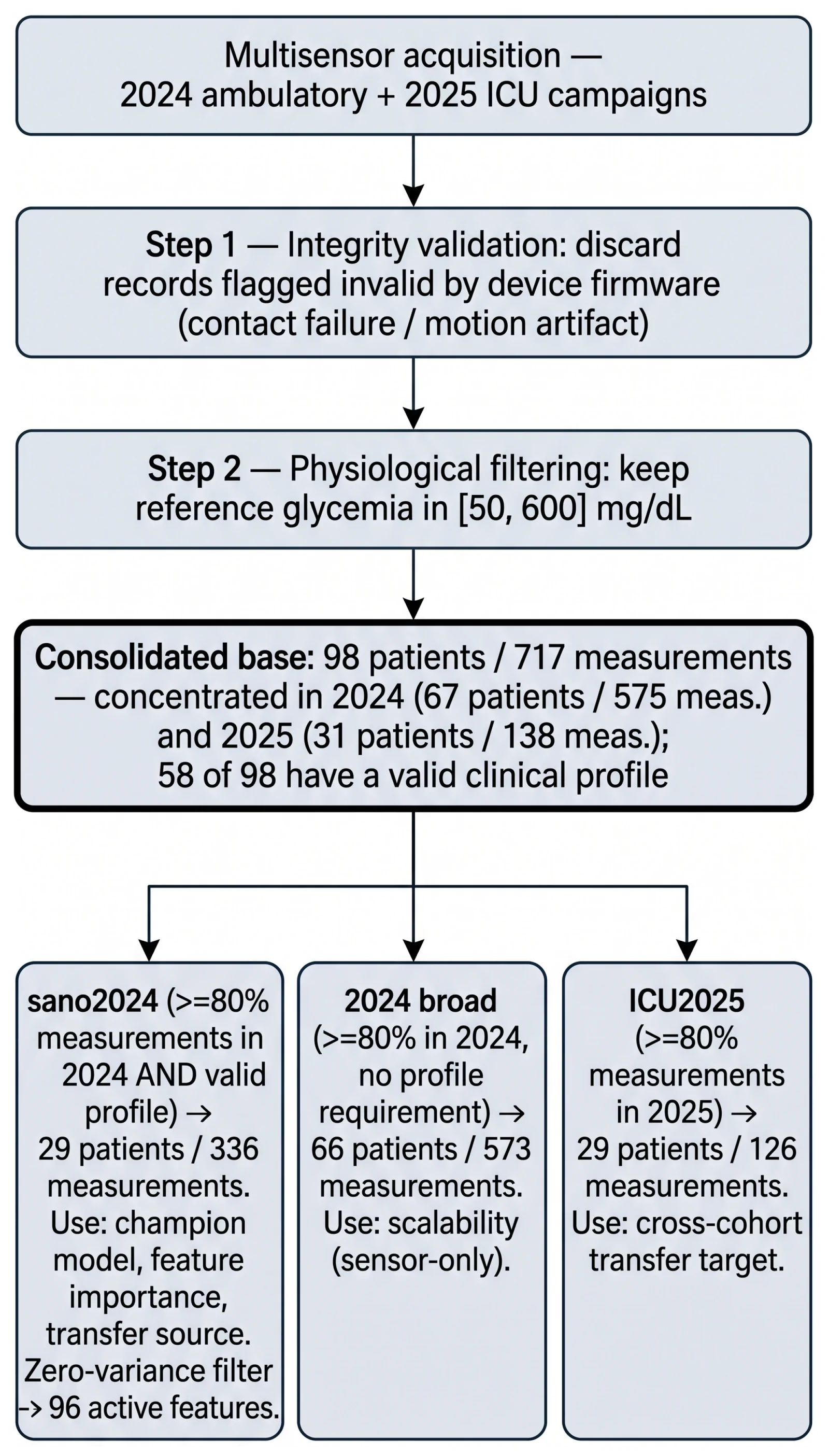
Cohort derivation (CONSORT-style). Integrity validation and physiological filtering ([50,600] mg/dL) of the multisensor acquisition yield the consolidated base (98 patients/717 measurements; concentrated in the 2024 ambulatory and 2025 ICU campaigns; 58 of 98 with a valid clinical profile). Three analysis cohorts are then derived by a temporal filter (≥80% of measurements in a given year) combined with the clinical-profile criterion: the curated sano2024 (29 patients/336 measurements; champion, importance and transfer source; zero-variance filtering leaves 96 active features), the sensor-only 2024 broad cohort (66/573; scalability), and ICU2025 (29/126; transfer target). The counts removed at each filter explain the differences between the raw campaign totals (67/575, 31/138) and the ≥80%-filtered cohorts (66/573, 29/126); sano2024’s 29 patients are the profile-valid subset of the 58.

Analysis cohorts. From the sanitized base, the analysis cohorts of Table 1 (with the full derivation traced in Figure 2) are derived by combining the temporal split with the clinical-profile integrity requirement. The curated ambulatory cohort sano2024 (29 patients with an integral profile and ≥80% of measurements in 2024) supports the champion model, the importance analysis and the transfer source. The broad 2024 cohort (66 patients, no profile requirement) is used only in the scalability test of the sensor-only model, and the ICU2025 cohort (29 patients admitted in 2025) is the transfer target. The preliminary architecture explorations (classification, SNN+SVR and the input-encoding study) were conducted on the full 98-patient base—which mixes both campaigns and relies on profile imputation for the 40 patients without a valid record—and are therefore reported only as a preliminary baseline.

Temporal abstraction. Unlike conventional ANNs that map static matrices, SNNs operate in the time domain. To represent this biological dynamic, the analog signal was abstracted into sequential sliding windows. Ablation analyses (window sizes T=5, T=10 and T=20) revealed the diminishing returns of temporal memory for this biosignal: overly wide windows introduced statistical noise and diluted the instantaneous glucose correlation, and at T=20 the requirement of continuous temporal sequences exhausted the viable windows per patient, precluding training. Therefore, the optimal abstraction window was set at T=5 discrete steps.

### 3.2. Validation Protocol: Walk-Forward Cross-Validation

**Figure 3:**
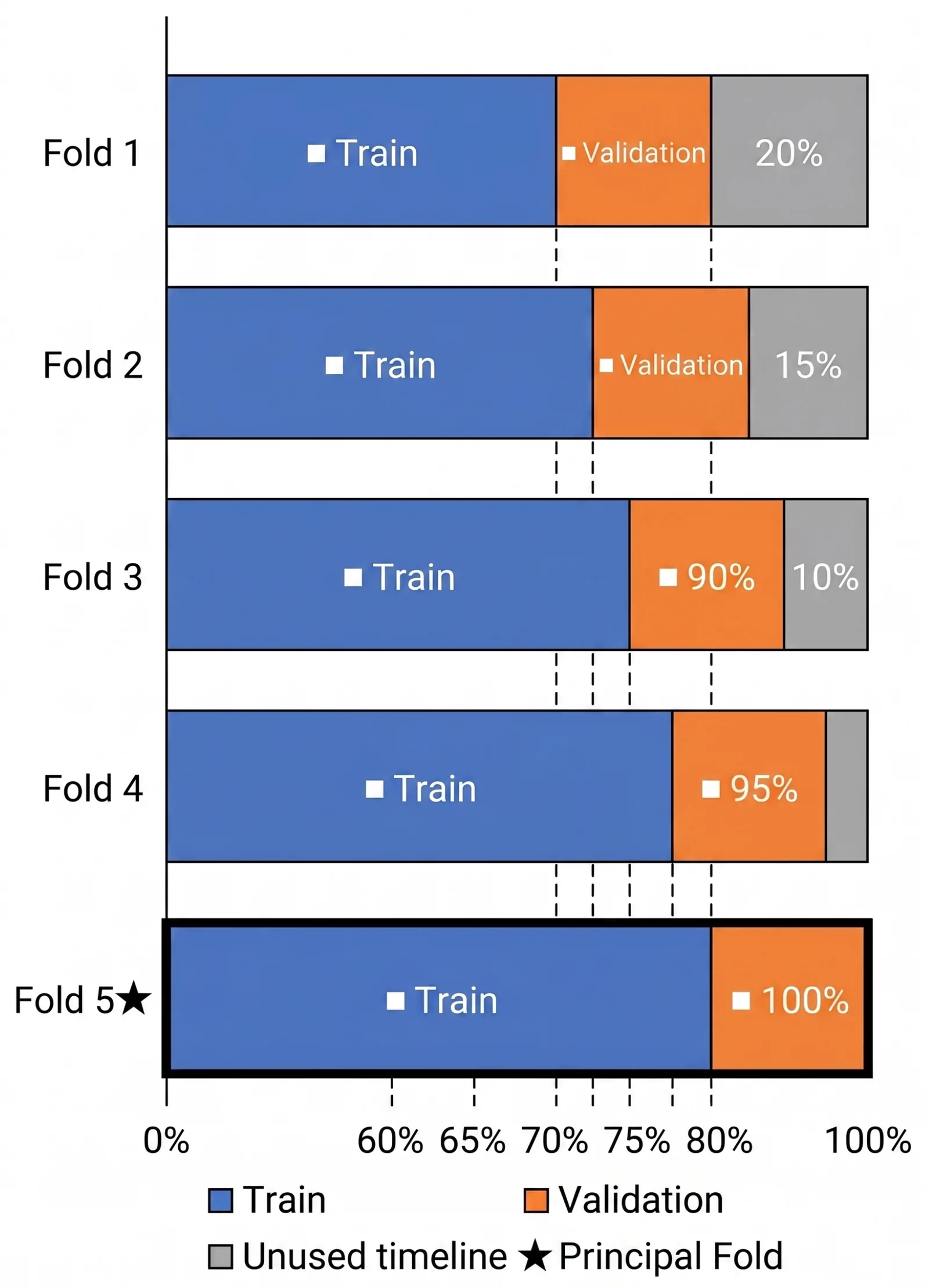
Five-fold walk-forward cross-validation scheme. Each bar represents a patient’s time series split into training (blue), validation (orange) and unused period (gray). Fold 5 (⋆), with an 80%/20% split, is the principal reported result; the others provide statistical robustness estimates. All patients are present in both the training and validation sets of each fold.

To assess the temporal robustness of the models and prevent any leakage of future data into training, we adopted a 5-fold walk-forward cross-validation protocol. In each fold *k*, the training set spans $\left[0,\;v_{k}\right)$ and validation spans $\left[v_{k},\;v_{k}+0.20\right)$ of each patient’s time series, with v={0.60,0.65,0.70,0.75,0.80}. Crucially, all patients appear in both sets of every fold, ensuring that the calibration one-hot is useful at inference. Fold 5 (the 80%/20% split) is reported as the principal result; the remaining folds provide robustness estimates (mean ± SD). The walk-forward scheme is illustrated in Figure 3.

### 3.3. Predictive Topologies Explored

Before consolidating the final end-to-end regression topology, two alternative approaches to the non-linear mapping of bioimpedance were investigated. Classification into discrete values: discretizing the glycemic spectrum into 10 and 25 mg/dL bins led to rapid overfitting, owing to the loss of the ordinal relation imposed by the CrossEntropy loss and the sample scarcity of boundary classes. Hybrid SNN+SVR architecture: using the SNN as a temporal feature extractor as proposed by Lee et al. [8], delegating the final prediction to an RBF-kernel SVR (C=100, γ=scale, ϵ=0.01), proved suboptimal, since the hidden-layer firing rates—interpreted in isolation from their accumulated integrative potential—lost the representational richness needed to distinguish abrupt variations. Finally, as a conventional non-spiking baseline, we trained a dense multilayer perceptron (MLP; flattened 5×195 window →128→64→1, ReLU) on the same fingerprint-calibrated inputs and the same walk-forward protocol, and additionally quantized its weights to int8.

### 3.4. The Neuron Model for Continuous Regression

**Figure 4:**
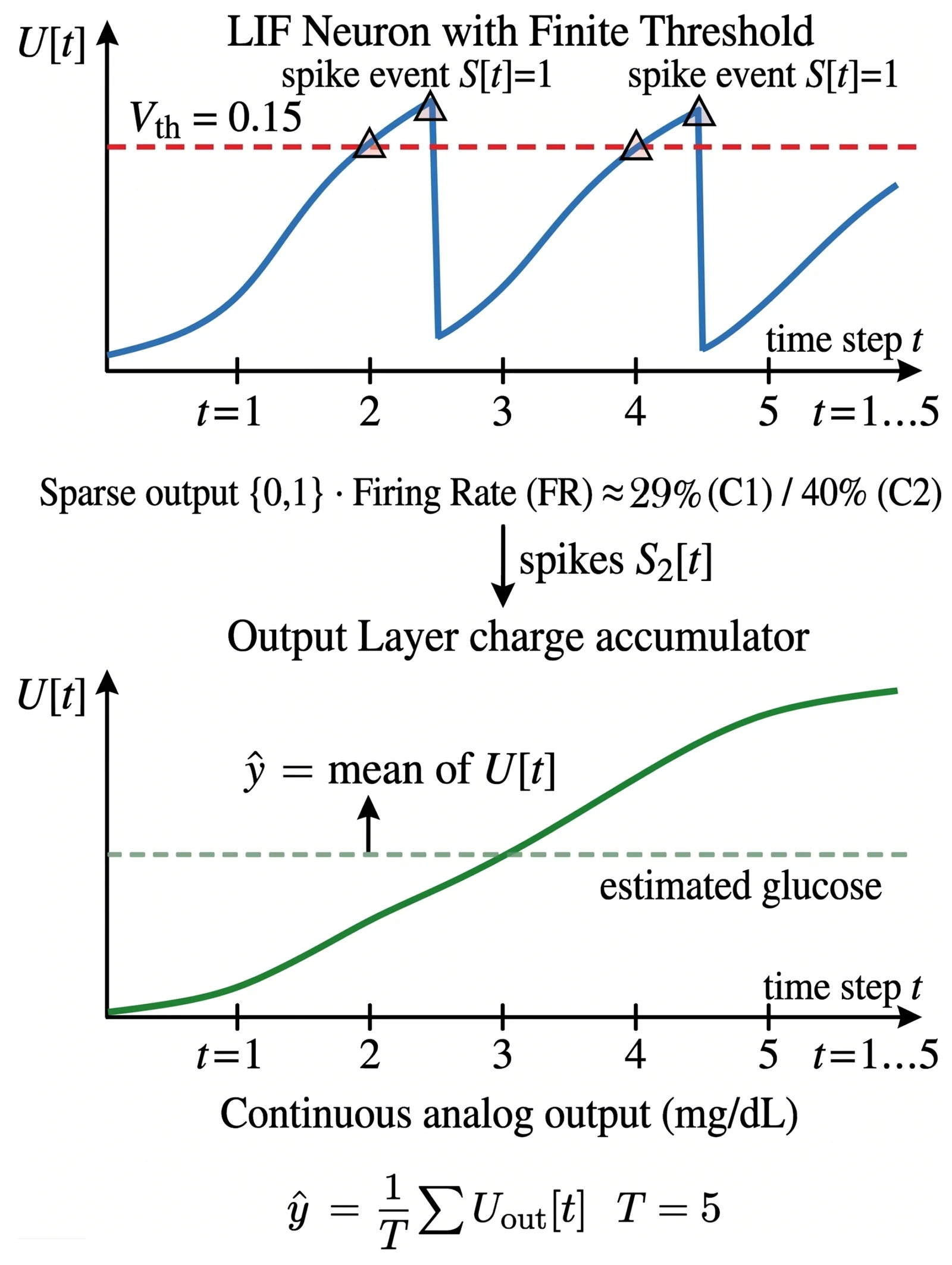
LIF neuron dynamics in the two architectural configurations. Top—hidden layers ($V_{th}=0.15$, β=0.80): the potential U[t] rises until it crosses the threshold, fires a spike (S[t]=1, orange triangles) and undergoes subtractive reset, producing sparse output. Bottom—output layer ($V_{th}\to \infty$, β=1.0, no reset): the potential accumulates continuously and its temporal mean $\hat{y}=\frac{1}{T}\sum U_{out}\left[t\right]$ directly represents the estimated glucose in mg/dL.

The fundamental element of the proposed architecture is the Leaky Integrate-and-Fire (LIF) neuron. The temporal dynamics of the membrane potential U[t] are governed by the decay equation:

(1) $$U\left[t\right]=\beta \;U\left[t-1\right]+\sum_{i}\left(W_{i}\cdot S_{i}\left[t\right]\right)-V_{th}\cdot S\left[t\right]$$

where β (fixed at 0.80) is the leak factor, $W_{i}$ is the synaptic weight, $S_{i}\left[t\right]$ is the event presence (continuous current or binary spike) at the input synapse, and the term $-V_{th}$·S[t] implements the subtractive reset after firing (S[t]=1). In the hidden layers the threshold is fixed at $V_{th}=0.15$. In the output layer the firing mechanism is disabled ($V_{th}\to \infty$, hence S[t]=0 for all *t*) and the leak is removed ($\beta_{\mathrm{out}}=1.0$, no leak), turning the neuron into a continuous charge accumulator; the temporal mean of its potential over the *T* steps directly represents the estimated glucose concentration. Figure 4 illustrates the membrane-potential behavior in both configurations. To isolate the contribution of this temporal accumulator, we ablated it against a direct linear readout—a single linear layer on the temporal-mean firing rate of the last hidden layer—keeping the spiking hidden layers, and thus the nonlinearity, identical (results in Section 4.3).

### 3.5. Input-Encoding Topologies

To assess the trade-off between clinical predictive accuracy and energy savings on edge operations, two main data-injection schemes were developed. Direct Injection (temporal spike): the normalized analog signal is continuously converted into current and processed step by step. This preserves the full signal resolution and maximizes accuracy, but requires multiply–accumulate (MAC) operations in the first synaptic layer. Sparse encoding (Rate-Coding): the analog value is replaced by the spike-emission probability through a Bernoulli distribution, turning the first hidden layer into a purely additive (AC) operation matrix—the ideal solution for energy-limited architectures. In the baseline comparison (T=5, no fingerprint), the stochastic evaluation was stabilized by averaging 30 Monte Carlo passes per sample. In the fingerprint variant (Section 3.7), the Straight-Through Estimator (STE) [18] was adopted: the gradient backpropagates through the Bernoulli operation as if the identity were applied ($\partial \hat{x}/\partial x=1$), removing the need for multiple samples during training. To compensate for the limited six-level resolution at T=5, the number of rate-coding steps was decoupled from the temporal window: each window step was subdivided into $T_{\mathrm{per}\text{\_}\mathrm{step}}=T_{\mathrm{rate}}/T$ microticks, with $T_{\mathrm{rate}}$ (the total rate-coding microticks per inference) ∈{25,50} evaluated by ablation.

Both topologies were first evaluated with patient identification via one-hot encoding, without explicit clinical data. The next investigation examines whether incorporating clinical-profile metadata reduces residual mean bias and improves individual accuracy.

### 3.6. Enrichment with the Patient Clinical Profile

Motivated by the hypothesis that anthropometric and clinical factors influence the bioimpedance–glycemia correlation, we investigated a complementary model that concatenates to the sensor vector the five clinical-profile features of Section 3.1: age, BMI, sex, diabetes type and the has-profile flag. Data were collected from 98 patients from Azambuja Hospital in Brusque (Brazil) using the eGluco3 device, which was developed by the research group at Santa Catarina State University. This experiment, like the input-encoding comparison (Direct Injection vs. Rate-Coding), operates on the 98-patient cohort with one-hot identification. The strategy expands the input vector from N=194 to N=199 dimensions and adds 640 trainable parameters. The importance of each feature was quantified by architecture-agnostic Permutation Feature Importance: each feature is shuffled individually in the validation set and the MAE degradation is measured against the baseline, with 10 repetitions per feature for statistical stability. Although the clinical profile improves accuracy, one-hot encoding remains a critical operational restriction: the model only recognizes patients present in the training set, precluding deployment on commercial devices with new users.

### 3.7. Patient Fingerprint Calibration

The clinical-profile and one-hot model assumes that the device recognizes the user—a critical limitation for embedded deployment since new patients require retraining. To overcome this, we investigated a dynamic calibration mechanism called the Patient Fingerprint: a vector $f_{k}\in R^{96}$ computed as the arithmetic mean of the patient’s first *K* sensor vectors in the training window,

(2) $$f_{k}=\frac{1}{K}\sum_{j=1}^{K}x_{j}^{\mathrm{sensor}},$$

where $x_{j}^{\mathrm{sensor}}$ denotes the 96 bioimpedance channels of the *j*-th chronologically ordered measurement. The vector $f_{k}$ is concatenated to every temporal step of the sliding window, expanding the input from $N_{\mathrm{base}}$ to $N_{\mathrm{aug}}=N_{\mathrm{base}}+96$ dimensions. Unlike one-hot, the fingerprint does not require the patient to be present in training; only *K* initial measurements are needed to personalize it.

Fingerprint evaluation, which consolidates the proposed final solution, adopts a stricter temporal filter than the previous experiments, requiring from each patient a continuous series long enough to compute the calibration vector under the walk-forward protocol (≥80% of measurements in 2024). This reduces the cohort to 29 patients (336 measurements) with dense temporal history. For a fair comparison, the reference one-hot model is retrained on this same 29-patient cohort, not on the 98-patient cohort used in the input-encoding study. Under this stricter filter, the has-profile flag becomes constant (zero variance, since all 29 patients hold a valid profile) and is dropped, while sex is evaluated and excluded for exhibiting negative permutation importance on this subset. Three active clinical features therefore remain—age, BMI and diabetes type—so the base windows hold 96(sensor)+3(clinical)=99 channels. In this protocol, walk-forward quantifies temporal robustness on known patients (present in both training and validation of each fold), whereas generalization to unseen individuals is assessed separately in the cross-cohort transfer (Section 3.8). The effect of *K* was studied by ablation (K∈{3,5,7,10}) in two variants: (a) windows holding the 99 base channels ($N_{\mathrm{aug}}=195$) and (b) windows restricted to the 96 sensor channels ($N_{\mathrm{aug}}=192$), removing the dependence on clinical records and simulating a purely sensor-based device. A complementary temporal analysis quantified the mean interval Δ*t* between the first and *K*-th measurement per patient, assessing the calibration latency in a realistic clinical scenario. We further characterized the fingerprint on the curated cohort along four axes: the metabolic context of the calibration window (reference glucose of the first *K* readings versus each patient’s own distribution), its temporal stability (cosine similarity between the fingerprint of the first and last *K* readings), its identity separability (between- versus within-patient distances of the standardized sensor signature), and the effect of alternative calibration strategies—first-*K* mean, whole-series centroid, and selection at a representative glycemic state—under the leave-patients-out protocol.

### 3.8. Cross-Cohort Transfer Evaluation

To assess generalization to distinct clinical environments, a cross-cohort transfer study was conducted. The sano2024 set (ambulatory diabetic patients) was used as the source domain; the ICU2025 set, with patients admitted to an intensive care unit in 2025, was used as the target domain. The experiment adopted two approaches: (i) training the fingerprint model directly on the ICU cohort with the same walk-forward protocol (scarce data—10 to 23 training windows per fold—required a minimum threshold of $N_{\mathrm{tr}}=5$ windows and an adaptive batch size); (ii) zero-shot transfer—full training on sano2024 without a train/validation split (220 windows), followed by direct evaluation on the ICU patients, aligning the feature space to that of sano2024 and reusing the glucose normalization scaler trained on sano2024 ([70,600] mg/dL).

To test generalization to unseen patients within the same domain—distinct from the cross-domain transfer above—we additionally performed a leave-patients-out (LOPO) evaluation on sano2024. Patients were partitioned into five groups; for each, the model was trained exclusively on the remaining patients (with early stopping on an internal split of the training patients, never the held-out ones) and then evaluated on the held-out patients, whose only patient-specific information was the fingerprint computed from their first *K* sensor readings (calibration, not training). Only windows whose target fell after the *K* calibration readings were scored, and the calibration depth was swept over K∈{3,5,7,10}.

### 3.9. Training and Implementation

The network is structured as densely connected layers with topology (194→128→64→1)—the base configuration with one-hot patient identification; the clinical-profile and Patient Fingerprint variants adjust only the input-layer dimension—interleaved with Layer Normalization (LayerNorm) blocks to stabilize the internal membrane-integration dynamics. The deployed Patient-Fingerprint model has input width N=195 (96 sensors + 3 clinical + 96 fingerprint channels); its parameter count follows directly from the layer sizes as ${\left(195\;\times \;128\;+\;128\right)}_{\mathrm{fc}1}+256_{\mathrm{LN}}+{\left(128\;\times \;64\;+\;64\right)}_{\mathrm{fc}2}+128_{\mathrm{LN}}+{\left(64\;+\;1\right)}_{\mathrm{fc}3}=33,793$, the LIF neurons adding none (β and $V_{th}$ fixed). The other widths are distinct baseline/ablation configurations, not the deployed model: N=194 is the one-hot baseline (96 sensors + 98 one-hot IDs), N=199 is the baseline with the 5 clinical enrichment features (Section 3.6), and N=192 is the sensor-only fingerprint (96 sensors + 96 fingerprint). Training relied on Backpropagation Through Time (BPTT). Since the binary activation has no continuous derivative, the Surrogate Gradient method was applied as a fast-sigmoid approximation (slope =15). The Adam optimizer was combined with the Smooth L1 (Huber) loss, which proved superior to the conventional mean squared error by damping the sparse variances inherent to sensor biology. A plateau-based learning-rate scheduler (ReduceLROnPlateau) with patience of 25 epochs and early stopping with patience of 60 epochs were used. Algorithm 1 formalizes the signal-propagation decisions of the proposed neuromorphic system.
**Algorithm 1** Regression processing in the proposed SNN**Require:** Biosignal matrix *X* of size T×N, threshold $V_{th}$  1:Initialize membrane potentials $U_{1},U_{2},U_{out}\leftarrow 0$  2:**for** t=1 **to** T=5 **do**  3:   **if** operation mode = Rate-Coding **then**  4:       $S_{in}\left[t\right]\leftarrow \mathrm{Bernoulli}\left(X\left[t\right]\right)$ {Stochastic binarization}  5:       $I_{1}\left[t\right]\leftarrow \mathrm{LayerNorm}\left(W_{1}\cdot S_{in}\left[t\right]\right)$ {AC operations}  6:   **else**  7:       $I_{1}\left[t\right]\leftarrow \mathrm{LayerNorm}\left(W_{1}\cdot X\left[t\right]\right)$ {MAC operations}  8:   **end if**  9:   $S_{1}\left[t\right],U_{1}\left[t\right]\leftarrow \mathrm{LIF}\left(I_{1}\left[t\right],U_{1}\left[t-1\right],V_{th}\right)$10:   $S_{1}\left[t\right]\leftarrow \mathrm{Dropout}\left(S_{1}\left[t\right],\;0.2\right)$11:   $I_{2}\left[t\right]\leftarrow \mathrm{LayerNorm}\left(W_{2}\cdot S_{1}\left[t\right]\right)$12:   $S_{2}\left[t\right],U_{2}\left[t\right]\leftarrow \mathrm{LIF}\left(I_{2}\left[t\right],U_{2}\left[t-1\right],V_{th}\right)$13:   $I_{out}\left[t\right]\leftarrow W_{out}\cdot S_{2}\left[t\right]$14:   $U_{out}\left[t\right]\leftarrow U_{out}\left[t-1\right]+I_{out}\left[t\right]$ {$\beta_{out}=1.0$, $V_{th}\to \infty$: leak-free accumulation}15:**end for**16:**return** $\frac{1}{T}\sum_{t=1}^{T}U_{out}\left[t\right]$ {Mean potential as predicted glucose}

### 3.10. Clinical Validation Criteria: ISO 15197:2013

Clinical conformity was assessed under two complementary criteria of ISO 15197 [9]. Criterion A (Section 6.3.3): at least 95% of results must meet a two-phase target: (i) for concentrations below 100 mg/dL, the absolute error must not exceed ±15 mg/dL; (ii) for concentrations ≥100 mg/dL, the percentage relative error must lie within ±15%. The Mean Absolute Relative Difference (MARD) is computed sample by sample:

(3) $$\mathrm{MARD}=\frac{1}{n}\sum_{i=1}^{n}\frac{|{\hat{y}}_{i}-y_{i}|}{y_{i}}\times 100\%$$

Criterion B—Consensus Error Grid (CEG, Annex C): evaluates the clinical impact of errors by risk zones. The standard requires that at least 99% of samples remain in Zones A+B.

### 3.11. Computational-Cost Quantification

The operational cost per inference was quantified for the deployed Patient-Fingerprint 195→128→64→1 architecture with T=5 steps (the one-hot baseline on the 98-patient cohort instead has N=194; all cost figures below refer to the deployed N=195 model), distinguishing MAC (multiply–accumulate) from AC (conditional-accumulation) operations. For Direct Injection, the first layer always executes 195×128×5=124,800 MACs, since the input is continuous; layers 2 and 3 execute ACs proportional to the measured firing rate. For Rate-Coding, the first layer executes only ACs (binary Bernoulli input). The hidden-layer firing rate was measured empirically by a forward pass over the fold-5 validation set, accumulating each layer’s spike tensors to obtain the mean fraction of neurons firing per time step. The cost model further accounts for the LayerNorm operations (which retain multiplications and are therefore not multiplication-free) and, for Rate-Coding, the per-microtick Bernoulli random-number generation (a software cost on microcontrollers without a hardware RNG); the resulting first-order energy is reported in the hardware trade-off analysis (Section 4.5).

### 3.12. Embedded Export and Portability

Since the premise of this work is autonomous edge inference, an export pipeline was developed to transcribe the offline-optimized neuromorphic model from PyTorch 2.11.0 (with the snnTorch 0.9.4 library) to bare-metal C/C++. A bare-metal implementation was chosen, decoupled from high-level commercial inference frameworks, to maximize computational efficiency and ensure hardware agnosticism. A concrete, representative candidate target of this class is an STMicroelectronics STM32F303CC (Arm Cortex-M4F at 72 MHz, 256 KB Flash, 40 KB SRAM)—the processing microcontroller of a next-generation wearable prototype currently in early bench testing, distinct from the eGluco3 device used for data acquisition (Section 3.1)—paired with a 2 KB FM24CL16B ferroelectric RAM (FRAM) and a 4 MB W25Q32 SPI flash; a companion ESP32-C3 handles only wireless connectivity, not inference. We use it below to ground the cost analysis with concrete figures, without implying a validated deployment. A transversal extraction routine reads the trained-model parameter dictionary (a .pth file), isolates the synaptic weight matrices and bias vectors of each (Linear and LayerNorm) layer, flattens them into single-precision (float) one-dimensional arrays, and automatically transcribes them into a unified header (.h) file. A key architectural decision was the deterministic allocation of all extracted parameters directly in non-volatile memory (Flash/ROM), preserving the scarce SRAM exclusively for the dynamic update of the membrane potentials U[t] and the multimodal input buffers. Concretely, the float parameter set (≈132 KB) fits within the STM32F303CC’s 256 KB internal Flash, from which it is read in place; the 2 KB FRAM stores the per-patient fingerprint vector $f_{k}$ for on-device recalibration, and the 4 MB W25Q32 is available for data logging. Int8 quantization, which would reduce the parameters to ≈33 KB, is therefore an optional optimization rather than a deployment requirement on this target, and it is most valuable on the ultra-constrained or neuromorphic hardware discussed below. Consistent with this scope, the present study reports the export pipeline and the first-order cost and energy profiles; the on-device execution and the instrumental measurement of latency and energy on the STM32F303CC are the object of a dedicated embedded study and are not claimed here.

## 4. Results and Discussion

Except for the preliminary classification and SVR explorations—conducted on the broad cohort of the initial design and cited only to contextualize the architectural choice—the results in this section refer to the 29-patient cohort with a real clinical profile, evaluated by 5-fold walk-forward cross-validation; fold 5 (80%/20%) is the principal result and the mean ± SD of the five folds is the robustness measure. The evaluation unit is the prediction window (a sequence of T=5 consecutive measurements) rather than the patient: each validation set gathers the windows contained in the final 20% of each patient’s series, so the principal fold sums ≈17 windows, fewer than the cohort’s patients (short series yield few or no validation windows). The cohort used is indicated per experiment: analyses that depend on clinical features are restricted to patients with a valid profile, whereas sensor-only analyses can exploit the broad cohort. Clinical conformity follows Criteria A and B of ISO 15197:2013.

### 4.1. Overview of the Approaches

Table 2 consolidates the performance of the patient-calibration strategies on the 29-patient cohort, from no calibration to the final fingerprint mechanism. Beforehand, two alternative formulations explored on the broad cohort were discarded for structural reasons. Classification into discrete bins (25 and 10 mg/dL) reached only 58% and 46% accuracy (MAE of 28.1 and 29.6 mg/dL): discretization discards the ordinal relation between bins—CrossEntropy treats adjacent classes as independent—and the sample-scarce extreme bins drive the model to collapse onto two or three central bins near the physiological mean. The hybrid SNN+SVR architecture, delegating prediction to an SVR fed by hidden-layer firing rates, regressed to a MAE of 38.8 mg/dL: isolated from the accumulated membrane potential, firing rates lose the representational richness needed to distinguish abrupt glycemic variations. In both cases, end-to-end LIF regression proved superior, justifying the architectural choice.

A conventional dense ANN baseline—an MLP on the same fingerprint-calibrated inputs and walk-forward protocol—reached a 5-fold MAE of 18.0±4.3 mg/dL (ISO A 68.2%, ISO B 100%), and int8 weight quantization left this essentially unchanged (18.0 mg/dL, a negligible loss). The end-to-end LIF regression is therefore not merely competitive but more accurate than the dense ANN (MAE 14.2 vs. 18.0 mg/dL), while remaining event-driven: the MLP executes 133,056 dense multiply–accumulate operations per inference with no path to multiplication-free execution, whereas the spiking model’s sparse activations map onto the add-native neuromorphic datapath quantified in the hardware trade-off analysis (Section 4.5). The SNN is thus justified on both accuracy and hardware-efficiency grounds.

Individual calibration is decisive: without it, the model is clinically unviable (MAE =35.9 mg/dL). One-hot encoding learns a per-patient identity vector (15.4 mg/dL) but requires the patient to be in the training set—unviable for new users. The Patient Fingerprint surpasses one-hot (14.2 mg/dL) without that restriction, deriving calibration from only *K* initial measurements, and constitutes the final solution. Its rate-coding variant trades accuracy for energy efficiency, as detailed below.

### 4.2. Final Solution: Accuracy, Energy and Embedded Feasibility

The proposed solution materializes in two embeddable fingerprint variants, identical in architecture (195→128→64→1, 33,793 parameters) and differing only in the first-layer encoding (Table 3). Direct Injection (K=3) preserves the analog signal resolution and delivers the lowest error (14.2±2.6 mg/dL MAE; 21.0±5.9 mg/dL RMSE in the walk-forward 5-fold CV, 20.1 mg/dL RMSE in the principal fold), the highest temporal stability and 100% ISO B, at the cost of keeping MAC operations at the input. Rate-Coding (K=5) replaces that layer with purely additive (AC) operations via Bernoulli binarization—an ideal target for ultra-low-power neuromorphic hardware—at the cost of +2.7 mg/dL MAE and a Criterion B of 98.8%, which falls below the 99% the standard requires—so, unlike direct injection (100%), the rate-coding variant does not formally meet Criterion B. This +2.7 mg/dL compares each variant at its own optimal *K* (injection K=3; rate-coding K=5). Isolating the encoding change at matched *K*, the rate-coding penalty over direct injection is +2.5 mg/dL at K=5 (16.9 vs. 14.5) and +5.1 mg/dL at K=3 (19.3 vs. 14.2); the headline value therefore closely tracks the encoding cost at rate-coding’s operating point (K=5), the larger K=3 gap reflecting rate-coding’s greater sensitivity to shallow calibration.

**Figure 5:**
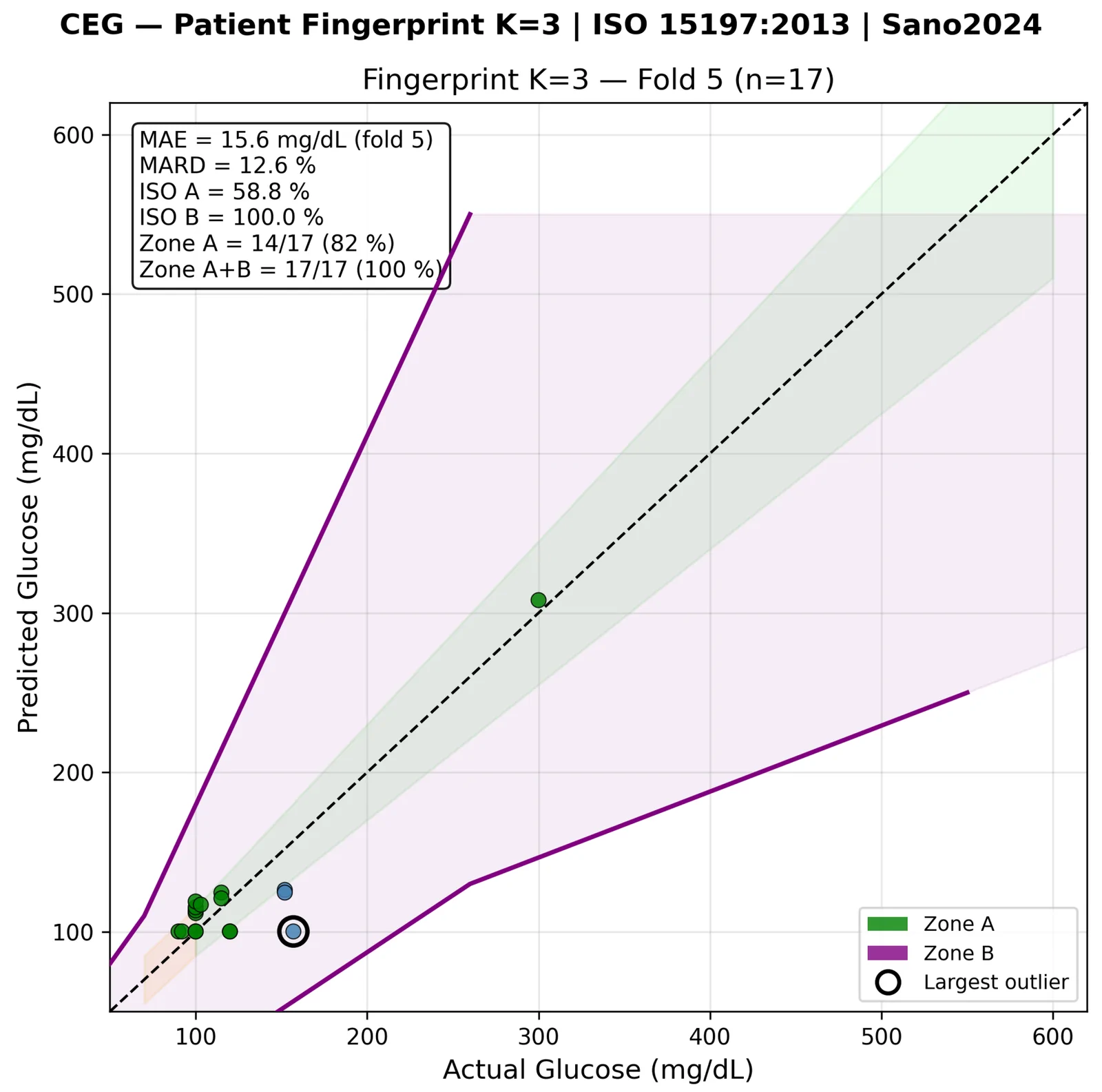
Consensus Error Grid (ISO 15197:2013) of the Direct Injection variant (K=3) in fold 5: 100% of samples in Zones A+B. On-panel metrics are single-fold (fold 5) values; Table 3 reports the 5-fold mean (14.2±2.6 mg/dL MAE).

**Figure 6:**
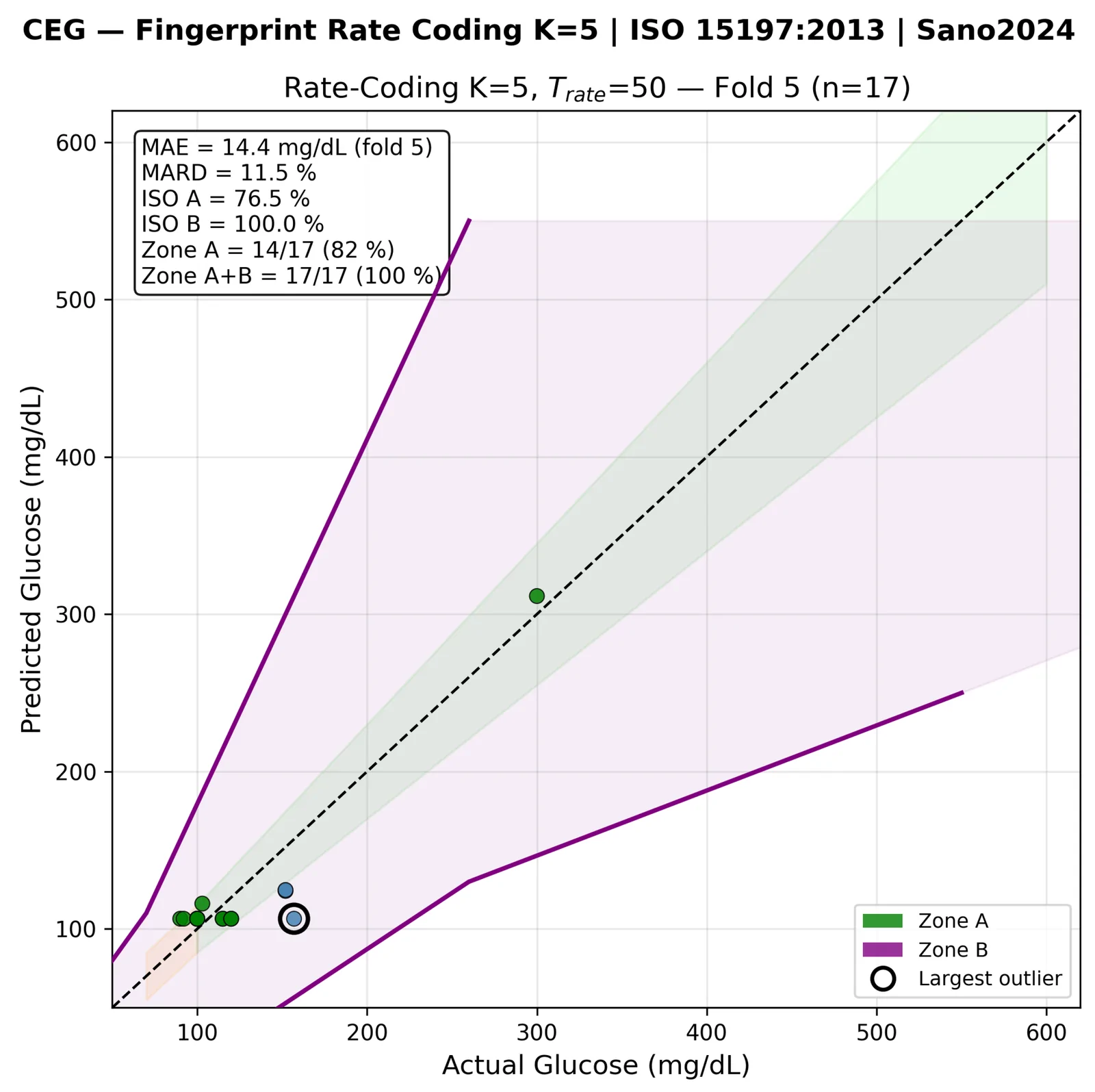
Consensus Error Grid (ISO 15197:2013) of the Rate-Coding variant (K=5, $T_{\mathrm{rate}}=50$) in fold 5. In this fold all 17 samples fall within Zones A+B (100%, MAE 14.4 mg/dL); across the five folds, however, the mean Criterion B is 98.8% (Table 3)—below the 99% normative threshold, so the rate-coding variant does not formally meet Criterion B—with a mean MAE of 16.9 mg/dL. On-panel metrics are this single fold (fold 5); Table 3 reports the 5-fold mean.

Figure 5 and Figure 6 show the Consensus Error Grid of each variant in fold 5.

A per-fold, per-range breakdown locates the rate-coding Criterion B shortfall precisely: the only fold below 100% is fold 2 (93.8%), where a single euglycemic window (reference 91 mg/dL, predicted 164 mg/dL) falls outside Zones A+B; every window in the sparsely covered extreme ranges (hypoglycemic and >250 mg/dL) stays within A+B. The 98.8% average is thus one isolated over-estimation in the target range, not a systematic failure at the extremes—and, with only ≈77 pooled validation windows, a single miss already represents ≈1.3%, underscoring the small-sample caveat.

Regarding cost per inference, Direct Injection executes 195×128×5=124,800 MAC operations in the first layer alone. Rate-Coding eliminates them entirely, rendering the datapath multiplier-free—a prerequisite for the most efficient neuromorphic accelerators. The high microtick count of the winning configuration ($T_{\mathrm{rate}}=50$, ten per measurement) is not arbitrary: since each normalized feature is encoded by its firing probability, few steps per measurement would offer very few discrete rate levels—only six {0;0.2;…;1.0} at T=5—insufficient to represent the dynamic range of bioimpedance; subdividing each measurement into ten microticks restores enough rate resolution to match direct injection. The counterpart is that processing 50 microticks (against 5 steps) multiplies the additive (AC) operations of the hidden layers by an order of magnitude, so the total operation count rises rather than falls. Whether trading multiplications for more additions yields a net energy gain depends on the target hardware—favorable on neuromorphic/integer hardware, where addition is the native, cheap operation, and marginal where the MAC–AC cost gap is small. The exact additive-operation count and energy balance depend on the firing rates measured in this 50-microtick regime and on the platform, and are quantified in the embedded implementation (future work). In both cases the float parameter set occupies ≈132 KB, fitting directly within the 256 KB internal Flash of the STM32F303CC (Cortex-M4F, 40 KB SRAM), and it is deployed at the reported accuracy without quantization; int8 quantization would further reduce it to ≈33 KB should a smaller target be required.

### 4.3. Factors of Influence

Temporal window. The T=5-step window proved the stability optimum: widening it to T=10 degraded the MAE to 62.2 mg/dL, and T=20 exhausted the continuous temporal windows available per patient, precluding training.

Output readout (accumulator vs. linear). Replacing the LIF output accumulator with a direct linear readout—a single linear layer on the temporal-mean firing rate of the last hidden layer, with identical spiking hidden layers—degraded the 5-fold MAE from 14.2±2.6 to 16.1±4.5 mg/dL and the RMSE from 21.0 to 25.1 mg/dL, and roughly doubled the between-fold variance, while both retained 100% ISO B. Because the accumulator is itself linear (β=1, pure summation), this gain is not additional nonlinearity—which resides in the hidden spiking layers and is unchanged here—but is temporal integration (memory): averaging the readout over the window stabilizes the estimate. The advantage lies in mean error and stability rather than the Criterion A hit-rate, which slightly favored the linear readout (78.4% vs. 64.3%); on balance, the accumulator is retained for its lower MAE/RMSE and temporal robustness.

Calibration latency. The mean interval between the first and *K*-th measurement grows with *K*: ≈0.5 days for K=3 (compatible with calibration at the first consultation) and ≈4.7 days for K=10, whose coverage drops to only 10 of the 29 patients—explaining the fingerprint degradation at high *K*.

Clinical features are context-dependent. The usefulness of demographic metadata is entirely conditioned on the context available to the model (Table 4). Without history (pointwise model, T=1), they become the most informative features—sex leads (ΔMAE =+11.9 mg/dL), followed by age (+5.3) and diabetes type (+3.9)—yet the pointwise model remains clinically inadequate (MAE =66.8 mg/dL; ISO B =84.1%). With temporal history, the clinical contribution collapses: diabetes type drops to +1.6 mg/dL, age becomes noise (+0.1) and sex flips sign (−1.4 mg/dL), consistent with a spurious shortcut in this small set (though the precise value and rank are not statistically robust at this sample size)—the reason for its exclusion from the deployed model. Consistently, the purely sensor-based fingerprint (no clinical data, K=5) matches the champion (14.2±2.4 mg/dL), confirming that sensor history renders the clinical record dispensable. We stress that, with only ≈17 validation windows, the permutation-importance magnitudes in Table 4 and Table 5 are indicative rather than definitive: we read only the qualitative pattern—clinical features become largely uninformative once temporal history is available, while patient identity dominates—and do not over-interpret sub-mg/dL differences or exact ranks. The reported ±values are between-fold standard deviations, the dispersion measure appropriate to the five walk-forward folds.

Patient identity dominates. In the temporal model, permutation importance is led by individual identity (Table 5): the two most informative one-hot features (+6.8 and +4.3 mg/dL) outweigh any single biomarker, motivating the per-patient calibration mechanism.

Cross-cohort generalization. Transfer to the intensive-care cohort (ICU2025, Table 6) delimits the application scope: zero-shot transfer collapses (MAE =117.6 mg/dL; ISO B =53.7%), and even direct training on the ICU cohort remains clinically insufficient (48.2 mg/dL), owing to data scarcity (10–23 windows per fold). The model is therefore specific to the ambulatory diabetic population on which it was trained.

Unseen-patient generalization (leave-patients-out). A stricter test of the new-user claim holds entire patients out of training and calibrates them only from their first *K* readings (Table 7). Accuracy is far below the intra-cohort walk-forward (MAE 14.2 mg/dL, where patients are represented in training) and improves markedly with calibration depth up to K=5 (MAE 65.9 mg/dL, $R^{2}=0.19$, ISO B 85.5%), then degrades as a larger *K* shrinks and shifts the test set (fewer post-calibration windows, longer-series patients only). Even at its best the model does not reach clinical grade for wholly unseen patients (ISO A well below 95%), and the between-fold variance is large. This indicates that the fingerprint is the correct calibration mechanism—more calibration readings help, and the operating point sits well above the collapse of the cross-domain transfer (117.6 mg/dL)—but that clinical-grade generalization to new individuals is bounded by the size of the training cohort (23 patients per fold), not by the mechanism, motivating a substantially larger multi-patient cohort as future work.

Fingerprint physiology and temporal stability. To understand this limit we characterized the fingerprint itself (Section 3.7). Three observations emerge. (i) Metabolic context: the first-*K* calibration readings are not a neutral snapshot—their reference glucose averages +24.8 mg/dL above each patient’s median (and is more dispersed), a bias that does not shrink with *K* (+22 to +24 mg/dL for K∈{3,…,10}), reflecting that monitoring typically begins in an elevated, unstable state. (ii) Temporal stability: the sensor signature drifts—the fingerprint of a patient’s first *K* readings and that of their last *K* share a mean cosine similarity of only 0.19—so a one-time calibration ages within the (short, median 2.7-day) series. (iii) Identity: despite this, at calibration time the fingerprint is a genuine individual signature (between-/within-patient distance ratio 1.23>1), although this separability erodes over the full series (0.80). Consistently, increasing the number of calibration readings improves unseen-patient accuracy through variance reduction (LOPO MAE 94.8→65.9 mg/dL from K=3 to K=5), whereas removing the bias does not: an unbiased whole-series centroid fingerprint is actually worse (LOPO MAE 121.7, being less discriminative), and calibrating at a representative glycemic state yields only a marginal gain in MAE (90.3) though it raises ISO A from 9.4 to 31.1%. The mechanism is therefore sound but limited here by cohort size and by a biased, drifting calibration window, pointing to periodic recalibration and representative-state calibration as concrete future directions.

Scalability without clinical data. By dispensing with the clinical record, the sensor-only fingerprint allows relaxing the integral-profile requirement and including all 2024 patients—expanding the cohort from 29 to 66 patients (573 measurements). On this broad cohort, direct injection keeps clinical conformity (ISO B ≈ 99%) and, in the training-densest fold, recovers the curated-cohort accuracy (MAE =14.2 mg/dL); robustness, however, degrades, with a mean MAE of 22.3±5.5 mg/dL—and the noisier rate-coding degrades further. This drop reflects the greater heterogeneity of the cohort and, possibly, the lower reliability of records from patients without a clinical profile, whose missing profile may correlate with less-curated acquisitions. The experiment shows that the method scales without any clinical metadata but benefits from a dense, curated cohort to reach its best accuracy.

The preceding results support the following discussion of the clinical scope, the hardware trade-off and the generalization limits of the solution.

### 4.4. Clinical Positioning and Comparison with the State of the Art

Clinically, the champion model attains a MAE of 14.2 mg/dL with 100% of samples in Zones A+B (Criterion B) across all folds, surpassing one-hot (15.4 mg/dL) without requiring the patient in training. Conformity to Criterion A, however, remains below the 95% normative threshold, precluding formal certification—a limitation attributed to the small validation set and the sparse coverage of extreme ranges (detailed in the Limitations section). The largest errors concentrate in glycemic transitions: patients whose profile during training differs from that observed at validation, which the model cannot anticipate without recalibration, thus motivating online recalibration as future work.

Compared with the predecessor work of Cruz Castañeda and Bertemes Filho [15], whose best regressor (cloud CatBoost) reports RMSE = 27.97 mg/dL and R^2^ = 0.31, the local SNN attains competitive performance, with an RMSE of 21.0±5.9 mg/dL in the walk-forward mean (20.1 mg/dL in the principal fold), even though evaluated over the full glycemic range [70–600], wider and more challenging than the predecessor’s narrow test distribution. Because the two evaluations are not range-matched, this comparison is indicative rather than controlled. Because R^2^ depends on the dispersion of each (different) test set, we do not treat the higher SNN R^2^ as evidence of superiority; RMSE and the deployment characteristics—not R^2^—form the basis of this comparison. Beyond accuracy, the SNN removes cloud dependence—whose latencies of up to 3737 ms and 11.46% failure rate under load are incompatible with critical continuous monitoring—and adds the normative ISO 15197 validation absent in the predecessor. The difference is also one of deployment: whereas CatBoost operates in the cloud, the SNN model occupies ≈132 KB and fits directly on the STM32F303CC (Cortex-M4F, 256 KB Flash/40 KB SRAM), enabling near-sensor inference. Table 8 summarizes the comparison.

### 4.5. The Hardware Trade-Off

To quantify the accuracy–hardware trade-off, we estimate the arithmetic energy per inference of the two embeddable variants (195→128→64→1). Operation counts use hidden-layer firing rates and the input spike probability measured on the fold-5 validation set—Direct Injection: ${\mathrm{FR}}_{1}=0.29$, ${\mathrm{FR}}_{2}=0.40$; Rate-Coding: $p_{\mathrm{in}}=0.52$, ${\mathrm{FR}}_{1}=0.44$, ${\mathrm{FR}}_{2}=0.41$—rather than assumed values. Per-operation energies follow Horowitz [19] at 45 nm: a multiply–accumulate costs 4.6/3.2 pJ and an addition 0.9/0.1 pJ (float/int). LayerNorm is counted separately because it retains multiplications, and the Rate-Coding Bernoulli sampling is charged as a software random-number-generation cost (the STM32F303CC has no hardware RNG). Table 9 reports the result.

By processing 50 microticks, Rate-Coding performs about 6.2× more total operations than Direct Injection. Whether this is a net gain depends entirely on the target’s multiply-to-add energy ratio: on a float microcontroller with an FPU (STM32F303CC class, ratio ≈5) Rate-Coding costs about 55% more energy, whereas on integer/neuromorphic hardware (ratio ≈32) it saves about 57%. This cross-over is the concrete criterion behind the encoding choice. Two observations sharpen it. First, LayerNorm is not multiplication-free and, executed once per microtick, becomes the dominant term on integer hardware; a multiplication-free normalization—or folding LayerNorm into the linear layers—would expose an ideal add-native datapath at ≈83 nJ per inference (−79% versus Direct Injection), which we flag as a promising direction for dedicated neuromorphic accelerators. Second, this is a first-order arithmetic estimate: it excludes memory-access and static (SoC) power, whose instrumental measurement on the target remains future work.

The central technical contribution is the mapping of the trade-off between accuracy and hardware architecture. Rate-Coding eliminates the 124,800 first-layer multiplications, rendering the datapath entirely multiplier-free—in the direction of neuromorphic accelerators such as SYNtzulA [5]—at the cost of +2.7 mg/dL MAE and an ISO B drop from 100% to 98.8%, which falls below the 99% Criterion B threshold—so the rate-coding variant does not formally meet Criterion B, whereas direct injection does. Notably, with $T_{\mathrm{rate}}=50$, this elimination does not reduce the total operation count: the hidden layers, processed over 50 microticks instead of 5, raise the additive operations enough for the total to exceed that of direct injection. The net energy benefit therefore depends on the target hardware—maximal on neuromorphic accelerators, where addition is the native, cheap operation, and tenuous where the multiply–add cost gap is small (as on the STM32F303CC, whose Cortex-M4F executes a single-cycle MAC, making multiply and add nearly equal in cost)—and is quantified to first order above (Table 9); its instrumental quantification is left to the embedded implementation. Still, the mapping provides a concrete design criterion: when the clinical margin is critical, direct injection is mandatory; when the target is multiplier-free hardware and the application can tolerate a Criterion B just below the normative threshold (98.8%), rate-coding is the choice.

The hidden-layer firing rate sits around 50%, with a bimodal pattern—silent and saturated neurons coexisting—structurally imposed by the LayerNorm preceding the LIF, which normalizes activations to zero mean. Attempts to make it sparser via adaptive thresholds reduced the firing rate at the cost of accuracy degradation, revealing a second trade-off between dynamic sparsity and predictive accuracy.

A practical edge-implementation question also remains. In the current flow, the firmware acquires the biosignals and transmits them; with direct injection, one would simply feed the normalized values into the first layer. Rate-coding, however, requires converting each feature into a Bernoulli spike train before that layer—a stochastic sampling (random-number generation and comparison per microtick) that, although removing synaptic multipliers, adds its own processing and randomness-generation cost on the microcontroller, repeated over the $T_{\mathrm{rate}}=50$ microticks. This encoding cost, absent in direct injection, must enter the total energy balance: the multiplier-free datapath advantage materializes only if spike generation is cheap enough (the STM32F303CC has no hardware random-number generator, so on this target the Bernoulli sampling falls back to a software generator)—for instance, via hardware pseudo-random generators or deterministic encoding schemes—reinforcing the need for instrumental evaluation in the embedded implementation.

### 4.6. Limitations and Future Work

The main limitation is the size and distribution of the dataset. ISO 15197:2013 itself requires, for system-accuracy evaluation, at least 100 samples distributed across prescribed glycemic ranges (with specific quotas for hypoglycemia ≤50 mg/dL and hyperglycemia >400 mg/dL). The principal-fold validation set holds only ≈17 prediction windows—not the 29 patient series, since each patient contributes windows only in its final 20%—with sparse coverage of the extreme ranges; even pooling the five folds, the total remains in the few dozens, about an order of magnitude below the normative minimum. Added to this is the inability to transfer to the ICU cohort (MAE 117.6 mg/dL zero-shot) whose bioimpedance is dominated by pathophysiological effects (edema, vasopressors) orthogonal to the ambulatory domain. Data-augmentation attempts only degraded performance, reinforcing that the bottleneck is the scarcity of real clinical data, not model capacity. The leave-patients-out analysis (Table 7) reinforces this from another angle: accuracy for wholly unseen patients improves with calibration depth (best at K=5) yet stays below clinical grade, a consequence of the small number of training patients rather than of the calibration mechanism.

Future work includes: (i) enlarging the cohort with coverage of the full physiological range and hyper/hypoglycemic episodes; (ii) implementing the bare-metal C/C++ model on the STM32F303CC microcontroller, with instrumental latency and consumption measurement; (iii) embedding the fingerprint protocol (computing $f_{k}$ on-device from the first *K* measurements), making the model genuinely agnostic to new patients, with periodic recalibration to counter the signature drift and calibration at a representative glycemic state to reduce the metabolic bias identified above; (iv) collecting ICU-specific data with domain-adaptation techniques.

## 5. Conclusions

This work proposed and evaluated a Spiking Neural Network architecture for non-invasive continuous glucose estimation from multimodal bioimpedance biosignals and is focused on the feasibility of execution on low-power embedded microcontrollers. The investigation systematically traversed the solution space—from problem formulation (end-to-end LIF regression, against discrete classification and hybrid SNN+SVR extraction) to the patient-calibration strategy (no calibration, offset, one-hot and Patient Fingerprint) and the input encoding (direct injection and rate-coding).

The Patient Fingerprint mechanism with K=3 measurements established itself as the final solution, reaching a MAE of 14.2±2.6 mg/dL and full conformity to Criterion B of ISO 15197 (100% of samples in Zones A+B) across all folds, surpassing the one-hot model without requiring the patient in training. The purely sensor-based variant matched this performance while dispensing with any clinical record, evidencing that sensor history implicitly encodes the individual physiology that demographic metadata only approximate—a finding whose dependence on temporal context was quantified by the importance analysis. A leave-patients-out analysis further showed the fingerprint to be a genuine individual signature whose accuracy improves with calibration depth, while delimiting the current scope: clinical-grade generalization—scaling with data rather than with the calibration mechanism—to wholly unseen patients is not yet reached on this 29-patient cohort and the cross-cohort transfer bounds the solution to the ambulatory population on which it was trained.

The central contribution to biomedical Edge AI is the quantitative mapping of the trade-off between clinical accuracy and computational cost: rate-coding eliminates the first-layer multiplications at the cost of +2.7 mg/dL MAE and a Criterion B that falls just below the normative 99% threshold (98.8%), providing objective criteria for selecting the encoding topology according to the hardware constraint. Together, these results position SNNs as a robust, clinically auditable and computationally lightweight alternative for glucose inference at the edge, paving the way for a bare-metal near-sensor implementation whose full energy advantage is realized on neuromorphic hardware. This potential broadens with the maturation of dedicated neuromorphic hardware. Platforms such as Intel Loihi 2, IBM TrueNorth, SpiNNaker and BrainScaleS, alongside compact biosignal-oriented accelerators such as SYNtzulA [5], natively execute spike dynamics with purely additive operations and a consumption on the order of a few picojoules per synaptic event. On such substrates, the multiplier-free rate-coding variant ceases to be a compromise and fully exploits the hardware efficiency, making continuous glucose estimation viable on autonomous ultra-low-power wearables.

## Figures and Tables

**Table 1:** Cohorts derived from the 98-patient/717-measurement base.

Cohort	Pat.	Meas.	Use in the Study
Base (preliminary)	98	717	Classification, SVR, encoding
sano2024	29	336	Champion; importance; transfer (source)
2024 broad (sensor)	66	573	Scalability
ICU2025	29	126	Transfer (target)

**Table 2:** Overview of patient-calibration strategies on the 29-patient cohort (mean ± SD, 5 folds). All use end-to-end LIF regression with direct injection, except the last (rate-coding).

Strategy	MAE (mg/dL)	MARD (%)	ISO A (%)	ISO B (%)
No calibration	35.9±11.7	24.6	32.3	95.1
Offset (N=7)	18.3±4.5	14.3	66.2	100
One-hot	15.4±3.3	11.4	79.4	100
Fingerprint (injection)	14.2±2.6	11.4	64.3	100
Fingerprint (rate-coding)	16.9±5.1	13.5	75.1	98.8

**Table 3:** The two embeddable fingerprint variants, each at its best *K* (mean ± SD, 5 folds; 195→128→64→1, 33,793 parameters).

Metric	Direct Injection (K=3)	Rate-Coding (K=5, $T_{r}=50$)
L1 encoding	Analog (MAC)	Bernoulli (AC)
MAE (mg/dL)	14.2±2.6	16.9±5.1
RMSE (mg/dL)	21.0±5.9	22.5±7.0
MARD (%)	11.4	13.5
R^2^	0.648	0.561
ISO A (%)	64.3	75.1
ISO B—CEG (%)	100	98.8
Time steps/microticks (*T*/$T_{\mathrm{rate}}$)	5	50

**Table 4:** Permutation importance (ΔMAE, mg/dL) of clinical features in two regimes—29-patient cohort.

Clinical Feature	No History (Pointwise)	With History (Temporal)
Sex	+11.9	−1.4
Age	+5.3	+0.1
Diabetes type	+3.9	+1.6
BMI	+0.9	+0.5

**Table 5:** Permutation importance in the temporal model (29-patient cohort, fold 5, $n_{\mathrm{rep}}=10$). ID: identity (one-hot); C: clinical; S: sensor.

Feature	Type	ΔMAE (mg/dL)
patient_24b…	ID	6.8
patient_394…	ID	4.3
maxled3	S	1.7
diabetes type	C	1.6
bmi	C	0.5
age	C	0.1
sex	C	−1.4

**Table 6:** Cross-cohort transfer—fingerprint K=3 trained on sano2024, evaluated on ICU2025.

Scenario	MAE (mg/dL)	ISO B (%)
Intra-cohort (sano2024)	14.2±2.6	100
Direct training on ICU	48.2±12.4	88.3
Zero-shot transfer sano→ICU	117.6	53.7

**Table 7:** Leave-patients-out (LOPO) on sano2024: patients held entirely out of training and calibrated only from their first *K* readings (five patient-grouped folds, aggregated over all held-out patients). For reference, the intra-cohort walk-forward reaches MAE 14.2 mg/dL/ISO B 100% (patients represented in training), and the cross-domain zero-shot transfer collapses to MAE 117.6 mg/dL/ISO B 53.7%.

*K*	Windows	MAE (mg/dL)	RMSE (mg/dL)	$R^{2}$	ISO A/B (%)
3	235	94.8	119.5	−0.14	9.4/74.9
5	220	65.9	101.3	0.19	36.8/85.5
7	191	85.8	121.9	−0.23	14.7/76.4
10	153	79.4	118.6	−0.35	19.0/74.5

**Table 8:** Comparison with the predecessor work of Cruz Castañeda and Bertemes Filho [15]. SNN values refer to walk-forward 5-fold cross-validation (mean) over the full glycemic range. The two studies are not range-matched (SNN over [70,600] mg/dL; predecessor over a narrower distribution), so R^2^ is listed for completeness only and is not used as evidence of superiority; RMSE is the primary basis of comparison.

Aspect	CatBoost (2024)	SNN (This Work)
Inference location	Cloud (CLL)	Edge (MCU)
R^2^ (5-fold mean)	0.31	0.65 (0.83 fold 5)
RMSE (mg/dL)	27.97	21.0±5.9
ISO 15197 validation	Not reported	Yes (Crit. A and B)
Latency under load	up to 3737 ms	local (no network)
Failure under load (50 users)	11.46%	—
Embedded footprint	n/a (cloud)	≈132 KB (float)

**Table 9:** First-order arithmetic energy per inference of the two embeddable variants (195→128→64→1). Firing rates were measured on the fold-5 validation set; per-operation energies follow Horowitz (45 nm). LayerNorm and Bernoulli RNG are listed separately. Energy excludes memory-access and static (SoC) power.

Quantity	Direct Injection (K=3)	Rate-Coding (K=5, $T_{r}=50$)
First-layer MAC	124,800	0
Accumulations (AC)	11,965	826,900
LayerNorm (mult/add)	2880/3840	28,800/38,400
Bernoulli RNG draws	0	9750
Measured firing rates	FR_1_ 0.29, FR_2_ 0.40	$p_{\mathrm{in}}$ 0.52, FR_1_ 0.44, FR_2_ 0.41
Energy, STM32F303CC (float, +RNG)	599 nJ	929 nJ (+55%)
Energy, neuromorphic (int, no RNG)	410 nJ	176 nJ (−57%)
Energy, ideal neuromorphic (int, no RNG/LN)	401 nJ	83 nJ (−79%)

## Data Availability

The data presented in this study are available on request from the corresponding author. The data are not publicly available due to privacy restrictions on patient clinical records. The model source code, trained weights, and the automatically generated C/C++ header (.h)—sufficient to reproduce the reported operation counts—are available from the corresponding author on reasonable request. They are not deposited in a public repository owing to intellectual-property considerations tied to ongoing patent activity.

## Abbreviations

The following abbreviations are used in this manuscript:

SNN	Spiking Neural Network
LIF	Leaky Integrate-and-Fire
MAC	Multiply–Accumulate
AC	Conditional Accumulation
MAE	Mean Absolute Error
MARD	Mean Absolute Relative Difference
CEG	Consensus Error Grid
BPTT	Backpropagation Through Time
STE	Straight-Through Estimator
MCU	Microcontroller Unit
ICU	Intensive Care Unit

## References

[1] Kim E., Kim Y. (2024). Exploring the potential of spiking neural networks in biomedical applications: Advantages, limitations, and future perspectives. Biomed. Eng. Lett..

[2] Choi S.H. (2024). Spiking neural networks for biomedical signal analysis. Biomed. Eng. Lett..

[3] Zhao S., Yang J., Wang J., Fang C., Liu T., Zhang S., Sawan M. (2023). A 0.99-to-4.38 μJ/class event-driven hybrid neural network processor for full-spectrum neural signal analyses. IEEE Trans. Biomed. Circuits Syst..

[4] Li H., Wang J., Zhao S., Tian F., Yang J., Sawan M. (2022). Real-time biosignal recording and machine-learning analysis system. Proceedings of the 2022 IEEE International Conference on Artificial Intelligence Circuits and Systems (AICAS).

[5] Martis L., Leone G., Raffo L., Meloni P. (2025). SYNtzulA: Open hardware for near-sensor SNN inference. IEEE Trans. Comput.-Aided Des. Integr. Circuits Syst..

[6] Yang J. (2020). A prediction scheme in spiking neural network (SNN) hardware for ultra-low power consumption. Proceedings of the 2020 International SoC Design Conference (ISOCC).

[7] Chu H., Yan Y., Zhou Y., Zhu Z., Huan Y., Zheng L., Zou Z. (2023). An energy-efficient and robust SNN classifier for LC-ADC sampled ECG signals. Proceedings of the 2023 8th International Conference on Integrated Circuits and Microsystems (ICICM).

[8] Lee C., Yang G., Baek J., Park Y., Cheon M., Park J., Park C. (2025). Regression model employing spiking neural network for bio-signal analysis with hardware integration. IEEE Access.

[9] (2013). In Vitro Diagnostic Test Systems—Requirements for Blood-Glucose Monitoring Systems for Self-Testing in Managing Diabetes Mellitus.

[10] Fang C., Shen Z., Tian F., Yang J., Sawan M. (2022). A compact online-learning spiking neuromorphic biosignal processor. Proceedings of the 2022 IEEE International Symposium on Circuits and Systems (ISCAS).

[11] Hens F., Dehshibi M.M., Bagheriye L., Tajadura-Jiménez A., Shahsavari M. (2025). LAST-PAIN: Learning adaptive spike thresholds for low back pain biosignals classification. IEEE Trans. Neural Syst. Rehabil. Eng..

[12] Gabriel S., Lau R.W., Gabriel C. (1996). The dielectric properties of biological tissues: II. Measurements in the frequency range 10 Hz to 20 GHz. Phys. Med. Biol..

[13] Maass W. (1997). Networks of spiking neurons: The third generation of neural network models. Neural Netw..

[14] Ponulak F., Kasinski A. (2011). Introduction to spiking neural networks: Information processing, learning and applications. Acta Neurobiol. Exp..

[15] Cruz Castañeda W.A., Bertemes Filho P. (2024). Improvement of an edge-IoT architecture driven by artificial intelligence for smart-health chronic disease management. Sensors.

[16] Sava R., Donati E., Indiveri G. (2023). Feed-forward and recurrent inhibition for compressing and classifying high dynamic range biosignals in spiking neural network architectures. Proceedings of the 2023 IEEE Biomedical Circuits and Systems Conference (BioCAS).

[17] Reddy B.S.S.B., Pidaparthi K., Kailath B.J. (2024). SNN with gradient-based backpropagation algorithm for ECG arrhythmia classification with LIF neuron and AdEx neuron. Proceedings of the 2024 28th International Symposium on VLSI Design and Test (VDAT).

[18] Bengio Y., Léonard N., Courville A. (2013). Estimating or propagating gradients through stochastic neurons for conditional computation. arXiv.

[19] Horowitz M. 1.1 Computing’s energy problem (and what we can do about it). Proceedings of the 2014 IEEE International Solid-State Circuits Conference (ISSCC).

